# Tumor Targeting by Conjugation of Chlorambucil with Zwitterionic Near-Infrared Fluorophore for Cancer Phototherapy

**DOI:** 10.3390/ijms232214093

**Published:** 2022-11-15

**Authors:** Gayoung Jo, Eun Jeong Kim, Hoon Hyun

**Affiliations:** 1 Department of Biomedical Sciences, Chonnam National University Medical School, Hwasun 58128, Republic of Korea; 2 BioMedical Sciences Graduate Program (BMSGP), Chonnam National University, Hwasun 58128, Republic of Korea

**Keywords:** chlorambucil, zwitterionic fluorophores, near-infrared fluorescence imaging, photothermal therapy, tumor targeting

## Abstract

Improving the tumor targeting of anticancer drugs to minimize systemic exposure remains challenging. The chemical conjugation of anticancer drugs with various near-infrared (NIR) fluorophores may provide an effective approach to improve NIR laser-induced cancer phototherapy. Towards this end, the selection of NIR fluorophores conjugated with hydrophobic anticancer drugs is an important consideration for targeted cancer photothermal therapy (PTT). In this study, a highly water-soluble zwitterionic NIR fluorophore (ZW800) was prepared to conjugate with a water-insoluble anticancer drug, chlorambucil (CLB), to improve tumor targeting, in vivo biodistribution, and PTT performance. The in vivo results using an HT-29 xenograft mouse model demonstrated that the CLB-ZW800 conjugate not only exhibited high tumor accumulation within 4 h after injection, but also showed rapid body clearance behavior for less systemic toxicity. Furthermore, the tumor tissue targeted by the CLB-ZW800 conjugate was exposed to 808 nm NIR laser irradiation to generate photothermal energy and promote apoptotic cell death for the effective PTT of cancer. Therefore, this study provides a feasible strategy for developing bifunctional PTT agents capable of tumor-targeted imaging and phototherapy by the conjugation of small molecule drugs with the versatile zwitterionic NIR fluorophore.

## 1. Introduction

Noninvasive photothermal therapy (PTT) generated by near-infrared (NIR) laser and photosensitizers have achieved great success regarding complete tumor ablation. Recently, many studies have focused on the development of more efficient PTT agents for enhanced cancer phototherapeutic efficacy [1,2,3]. Ideal PTT agents should meet the criteria for potential clinical utility: (i) strong NIR absorption to improve deep tissue penetration of NIR light, (ii) good biocompatibility, water solubility, and stability in biological systems, (iii) high photothermal conversion efficiency, and (iv) preferential tumor accumulation and apparent body clearance. Among the different types of PTT agents reported previously, commercially available heptamethine cyanine dyes, such as Cy7 [4], IRDye 800CW [5], IR-780 [6], IR-783 [7], IR-786 [8], and IR-808 (also called MHI-148) [9] have been widely used for tumor-targeted imaging and phototherapy.

ZW800-1, a zwitterionic (net neutral) heptamethine indocyanine fluorophore, has been previously highlighted as an excellent NIR fluorescent contrast agent with remarkable optical and physicochemical properties, including a high molar extinction coefficient and quantum yield, as well as good water solubility and stability compared to the clinically available indocyanine green and commercial NIR fluorophores [10,11]. Moreover, ZW800-1 has significant advantages for in vivo biomedical applications, because it contains no serum protein binding and exhibits rapid renal excretion from the body, ultralow nonspecific tissue/organ uptake, and a higher target-to-background ratio after conjugation with various targeting ligands [12,13]. Based on the superior optical properties and in vivo performance of ZW800-1, the ZW800-1 conjugates armed with tumor-targetable ligands can be utilized for NIR fluorescence-guided photothermal cancer treatment.

Chlorambucil (CLB), 4-{4-[bis(2-chloroethyl)amino]phenyl}butyric acid, is a well-known nitrogen mustard typically used as an anticancer drug in the treatment of leukemia, as well as breast, lung, and ovarian cancers. Since CLB is an alkylating agent which interferes with gene expression and inhibits DNA replication [14,15,16,17], CLB as a cytostatic drug is highly effective for the treatment of various solid tumors. However, the use of CLB is limited by its lack of specificity towards targeting cancer cells and its poor cellular permeability. Additionally, CLB shows low bioavailability and poor pharmacokinetics due to its poor water solubility and rapid degradation in the plasma, leading to adverse side effects and uncertain therapeutic efficacy [18]. To overcome these limitations, CLB has been mainly conjugated to various kinds of amphiphilic polymers, forming nanoparticles to improve its water solubility and bioavailability [19,20,21].

**Scheme 1:**
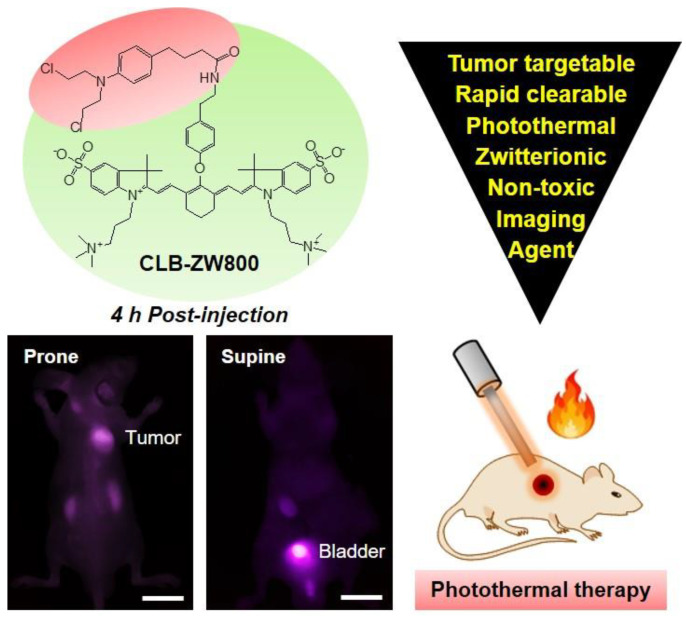
Schematic representation of the CLB-ZW800 conjugate used for tumor-targeted imaging and PTT. The tumor-targetable and rapidly body-clearable CLB-ZW800 conjugate provides a promising strategy for utilizing the hydrophobic drugs for various therapeutic purposes. Scale bars = 1 cm.

A simple and feasible approach to enhance tumor-selective accumulation and to prevent nonspecific tissue/organ uptake of the highly cytotoxic and hydrophobic CLB is to select the right NIR fluorophore with nontoxic, hydrophilic, and conjugatable features. Based on the optical and physicochemical properties of the conjugatable NIR fluorophores, the ZW800-1 is the best NIR fluorophore, especially in terms of hydrophobicity, to overcome the limitations of CLB (Scheme 1). In this study, the water-insoluble CLB was conjugated to a highly water-soluble zwitterionic NIR fluorophore (ZW800) by a tyramine linker forming an amide bond, hereafter referred to as CLB-ZW800, to improve the in vivo performance and targeted cancer PTT. The newly designed CLB-ZW800 conjugate provides a promising strategy for utilizing the various kinds of hydrophobic anticancer drugs for tumor-targeted drug delivery and imaging, as well as phototherapy, thereby acting as a multifunctional phototherapeutic agent.

## 2. Results

### 2.1. Synthesis and Characterization of CLB-ZW800 Conjugate

**Figure 1:**
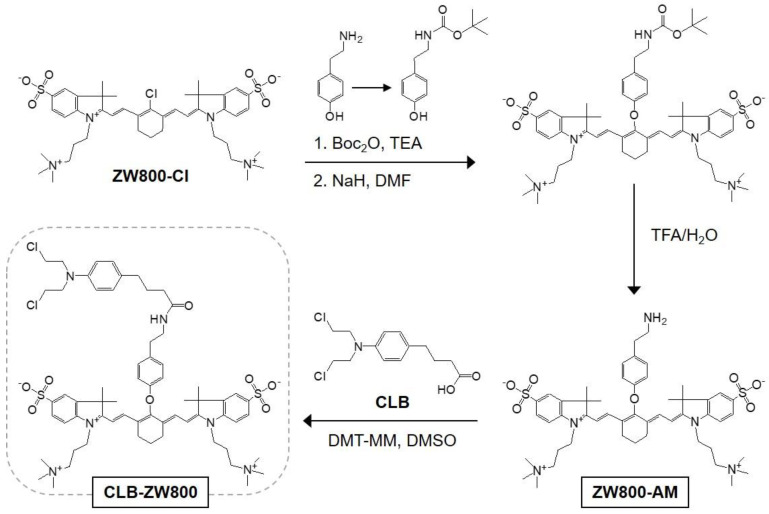
Synthetic scheme of ZW800-AM and CLB-ZW800 conjugate. The amine-functionalized zwitterionic NIR fluorophore ZW800-AM was prepared using the chloro-substituted ZW800-Cl via a nucleophilic displacement reaction of Boc-protected tyramine. After Boc deprotection, ZW800-AM can be covalently conjugated with CLB by forming the amide bond in a condensation reaction.

As an initial step, the original chemical structure of the zwitterionic NIR fluorophore, called ZW800-1, should necessarily be modified with the amine-functionalized structure of ZW800-AM, which can then be utilized for CLB conjugation. The amine-functionalized ZW800-AM was previously designed for conjugation with carboxylate-functionalized molecules, and it also exhibited excellent optical and physicochemical properties, as well as in vivo performance, including rapid renal clearance behavior similar to that of the ZW800-1 [22,23]. ZW800-AM was synthesized by a substitution reaction between an intermediate form of ZW800-Cl and amine-functionalized tyramine (Figure 1). The ZW800-Cl was prepared via a condensation reaction between the Vilsmeier−Haack reagent and zwitterionic indolium salts, based on the current good manufacturing practices (cGMP)-compatible synthetic procedure developed by Choi et al. [10,11]. Subsequently, a free primary amine in the tyramine structure was temporarily protected by the Boc group before use in the substitution reaction to prevent an unwanted reaction. After completion of Boc-protection, the phenoxide ion of tyramine could be linked to the meso-carbon of the heptamethine core of ZW800-Cl by using sodium hydride to increase the nucleophilicity of the protonated oxygen. Finally, a solution of TFA and water was used to remove the Boc group of tyramine, and then CLB could be conjugated with the amine-functionalized ZW800-AM via a condensation reaction in the presence of a coupling agent.

**Figure 2:**
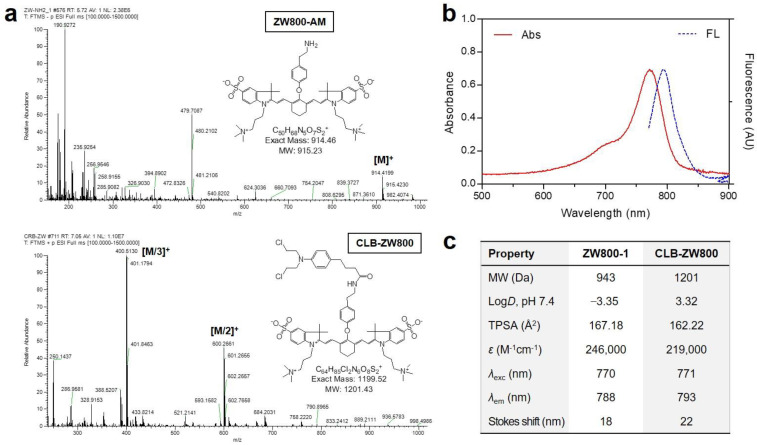
(**a**) Mass spectra of the ZW800-AM and CLB-ZW800 conjugate, (**b**) absorbance and fluorescence emission spectra of the CLB-ZW800 conjugate, (**c**) physicochemical and optical properties of the CLB-ZW800 conjugate compared to that of the first designed zwitterionic NIR fluorophore ZW800-1. Optical measurements were performed in PBS at pH 7.4. In silico calculations of the distribution coefficient (log*D* at pH 7.4) and topological polar surface area (TPSA) were performed using the Marvin and JChem calculator plugins (ChemAxon).

After purification by a preparative HPLC system, the molecular weights of the ZW800-AM and CLB-ZW800 conjugate were successfully confirmed by mass spectrometry (Figure 2a). The optical properties of the well-identified CLB-ZW800 conjugate were measured in PBS at pH 7.4. The absorption and fluorescence emission spectra of CLB-ZW800 displayed in the NIR spectral region peaked at 771 and 793 nm, respectively (Figure 2b). This indicates that CLB-ZW800 can be utilized for in vivo PTT applications combined with the NIR laser irradiation. Additionally, in silico calculations of the physicochemical properties, such as hydrophobicity (log*D*) and polarity (TPSA), were performed using JChem (ChemAxon) (Figure 2c). CLB-ZW800 showed increased hydrophobicity (log*D* = 3.32), owing to the water-insoluble CLB moiety, and maintained polarity (TPSA = 162.22 Å^2^) compared to that of ZW800-1 (−3.35 and 167.18 Å^2^, respectively). Although the increased hydrophobicity of CLB-ZW800 contributed slightly less to the molar extinction coefficient (*ε* = 219,000 M^−1^cm^−1^) of ZW800-1 (*ε* = 246,000 M^−1^cm^−1^), the highly water-soluble zwitterionic fluorophore could contribute to compensate for the poor water solubility of CLB. Hence, this CLB-ZW800 conjugate is a good combination of highly hydrophobic and hydrophilic properties to improve the solubility of water-insoluble CLB, compared to many types of commercially available hydrophobic NIR fluorophores. Moreover, the physicochemical characteristics of CLB-ZW800 may help to overcome the limitations of CLB, including low tumor selectivity, poor plasma stability, and systemic toxicity.

### 2.2. In Vitro Cytotoxicity and Cellular Uptake

**Figure 3:**
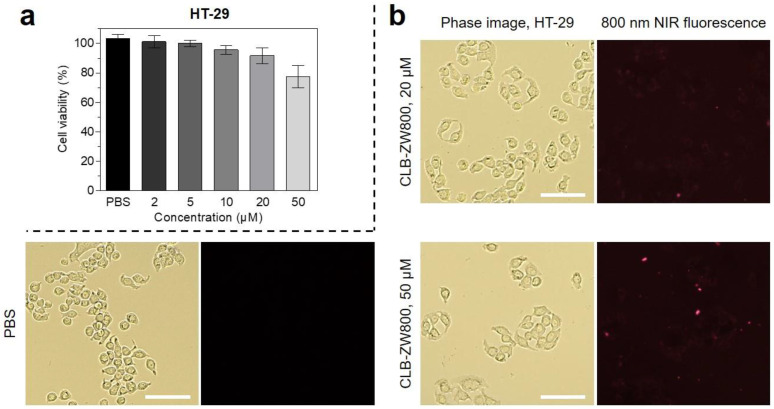
(**a**) Cell viability assay of the CLB-ZW800 conjugate using HT-29 cancer cells. The percentage of cytotoxicity was determined after 24 h of treatment with various concentrations of the CLB-ZW800 conjugate. (**b**) Live cancer cell binding of the CLB-ZW800 conjugate in the HT-29 cells 24 h after treatment with various concentrations of CLB-ZW800. The phase contrast and NIR fluorescence images of the cell line were obtained using 20 and 50 µM of CLB-ZW800, or PBS alone as a control. Images are representative of N = 3 independent experiments. All NIR fluorescence images have identical exposure times and normalization. The 800 nm NIR fluorescence was indicated by the red color. Scale bars = 100 μm.

In vitro cytotoxicity testing was performed using the MTT assay with various CLB-ZW800 concentrations (2, 5, 10, 20, and 50 μM) in HT-29 cancer cells for 24 h (Figure 3a). The CLB-ZW800 conjugate exhibited no significant cytotoxicity to the HT-29 cancer cells treated with the concentrations of 2–20 μM, and only a small cytotoxicity was observed at the 50 μM concentration. This suggests that the cytotoxicity of CLB is definitely compensated by the zwitterionic fluorophore moiety of CLB-ZW800, owing to its high water solubility and good biocompatibility, as reported previously [24]. In addition, the intracellular uptake of CLB-ZW800 was observed by the NIR fluorescence microscope system after 24 h of incubation with various CLB-ZW800 concentrations (2–50 μM) in the HT-29 cancer cells (Figure 3b). Unexpectedly, weak or no fluorescence signals in the cancer cells were detected in all treatment groups, even at the high CLB-ZW800 concentrations of 20 and 50 μM. Similar to the result of the cytotoxicity assay, a certain decrease in the number of viable cells was observed in the group treated with the 50 μM concentration of CLB-ZW800. This result indicates that the zwitterionic fluorophore moiety of CLB-ZW800 may act to prevent the permeation of the cell membrane, which is the well-known characteristics of ZW800-1 reported previously [24]. Moreover, the high polarity (TPSA = 162.22 Å^2^) of CLB-ZW800 is another important factor affecting membrane permeability, because the TPSA value greater than 140 Å^2^ tends to be poor at permeating cell membranes, according to the physicochemical property guidelines of Lipinski’s rule [25]. Thus, the zwitterionic property of CLB-ZW800 could contribute to improving the water solubility and cytocompatibility of CLB.

### 2.3. Time-Dependent In Vivo Tumor Imaging and Biodistribution

**Figure 4:**
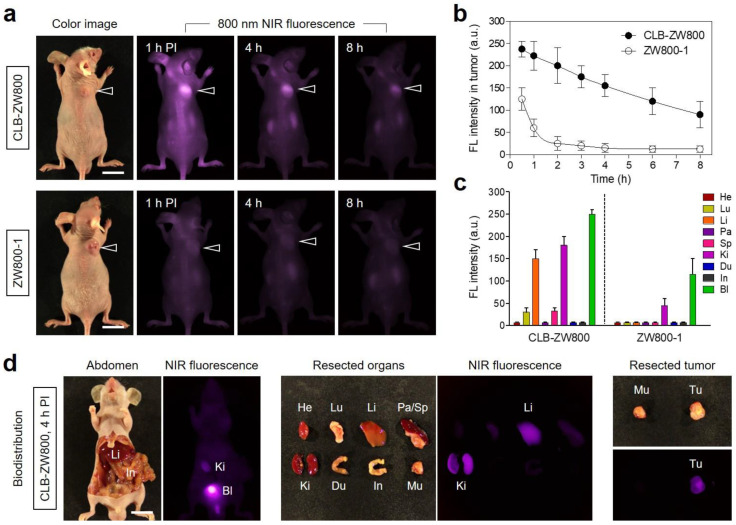
In vivo HT-29 tumor targeting efficiency and biodistribution of the CLB-ZW800 conjugate. (**a**) NIR fluorescence imaging 8 h after injection of CLB-ZW800 and ZW800-1. (**b**) Time-dependent fluorescence intensities at the tumor sites targeted by CLB-ZW800 and ZW800-1. (**c**) Quantitative fluorescence analysis of intraoperative dissected organs 4 h after injection of CLB-ZW800 and ZW800-1. (**d**) Biodistribution, resected major organs, and harvested tumor tissue imaged 4 h after injection of CLB-ZW800. The tumor sites are indicated by arrowheads. Abbreviations: Bl, bladder; Du, duodenum; He, heart; In, intestines; Ki, kidneys; Li, liver; Lu, lungs; Mu, muscle; Pa, pancreas; Sp, spleen; Tu, tumor; PI, post-injection. Scale bars = 1 cm. Images are representative of N = 3 independent experiments. All NIR fluorescence images had identical exposure times and normalization.

The in vivo tumor-specific accumulation and biodistribution of CLB-ZW800 were investigated using an HT-29 xenograft mouse model to confirm whether CLB-ZW800 could be used for the PTT application. CLB-ZW800 or ZW800-1 alone as a control group were administered intravenously into the tumor-bearing mice. The mice in each group were continuously imaged for 8 h using a real-time NIR fluorescence imaging system (Figure 4a). As expected, the ZW800-1 alone showed no significant accumulation in the tumor and rapid renal clearance in the body within 8 h after injection, which is typical of the in vivo behavior of ZW800-1 shown in the previous reports [10,11]. Unlike ZW800-1 alone, the CLB-ZW800 conjugate exhibited high tumor accumulation at 1 h post injection, and the fluorescence signal at the tumor site gradually decreased until 8 h after injection, accompanied by rapid body clearance behavior (Figure 4b). This demonstrates that the CLB moiety of the CLB-ZW800 conjugate played an important role in the tumor-specific accumulation. Moreover, the zwitterionic fluorophore moiety of CLB-ZW800 contributed to improving body clearance without nonspecific background uptake. Thus, the PTT application can be conducted at 1 h post injection of CLB-ZW800 to maximize the photothermal effect and induce tumor necrosis. Additionally, the biodistribution of CLB-ZW800 was identified by comparing the fluorescence signals of the main organs harvested from mice 4 h post injection (Figure 4c,d). CLB-ZW displayed fluorescence signals mainly in the liver and kidneys, as well as in the tumor tissue, with a the strong fluorescence signal in the bladder, indicating that CLB-ZW800 was eliminated from the body predominantly by the renal route.

### 2.4. In Vitro and In Vivo Photothermal Effects

**Figure 5:**
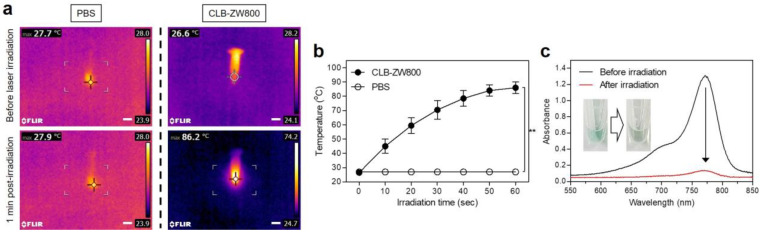
(**a**) In vitro thermal images of the CLB-ZW800 solution (10 μg/100 μL in PBS; 100 μM concentration is equivalent to a single dose of 0.48 mg/kg) and PBS alone (100 μL) exposed to an 808 nm laser (1.1 W/cm^2^) for 1 min. The maximum temperature was automatically recorded using an infrared thermal camera as a function of the irradiation time. Scale bars = 1 cm. (**b**) Temperature changes in the solutions in each sample were monitored during 1 min of laser irradiation. Data are expressed as mean ± S.D. (*n* = 3); ** *p* < 0.01. (**c**) Photostability of the CLB-ZW800 solution under laser irradiation. The absorbance changes were measured before and after 1 min of laser irradiation. The inset shows the color change of the CLB-ZW800 solution before and after laser irradiation. Images are representative of N = 3 independent experiments.

The photothermal conversion capability of CLB-ZW800 dissolved in PBS was investigated through a 1 min exposure to 808 nm laser irradiation (1.1 W/cm^2^). The temperature changes were directly monitored using an FLIR^®^ thermal imager. The power density of the 808 nm laser was optimally adjusted to avoid unnecessary tissue damage generated only by laser power, without the photothermal agent, as reported previously [26]. The NIR laser-induced temperature of the CLB-ZW800 solution immediately increased from 26.6 to 86.2 °C during the 1 min of laser irradiation, whereas the temperature of PBS alone displayed little change (from 27.7 to 27.9 °C) under the same conditions (Figure 5a). The NIR laser-induced temperature of CLB-ZW800 promptly increased up to ~70 °C during the first 30 s of laser irradiation, and finally reached up to ~86 °C during the next 30 s of irradiation (Figure 5b). This result demonstrates that CLB-ZW800 has an excellent photothermal conversion capability and can be used for in vivo photothermal cancer treatment. To check the photostability of CLB-ZW800, the absorbance of CLB-ZW800 was measured before and after 1 min of laser irradiation (Figure 5c). As expected, the absorbance of the CLB-ZW800 solution dramatically decreased after 1 min of laser irradiation. The color of the CLB-ZW800 solution was also changed from green to yellowish green under 1 min of laser irradiation. This indicates that the heptamethine skeleton of NIR fluorophores can be easily damaged under the NIR laser irradiation with a certain power density after showing the photothermal conversion effect.

**Figure 6:**
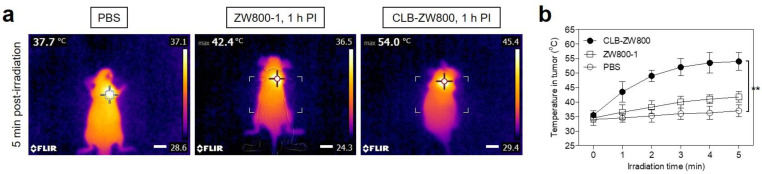
(**a**) Whole-body thermal images of tumor-bearing mice 1 h after injection of PBS, ZW800-1, or CLB-ZW800 upon exposure to 808 nm laser irradiation (1.1 W/cm^2^) for 5 min. Scale bars = 1 cm. (**b**) Temperature changes at the tumor sites in each treatment group were monitored during 5 min of 808 nm laser irradiation. Data are expressed as mean ± S.D. (*n* = 3); ** *p* < 0.01. Images are representative of N = 3 independent experiments.

Importantly, the in vivo PTT capability of CLB-ZW800 was further investigated using an HT-29 tumor-bearing mouse model under NIR laser irradiation. CLB-ZW800, ZW800-1, or PBS were intravenously injected into the HT-29 xenograft mouse model 1 h before laser irradiation. Subsequently, the tumor tissues were exposed to 808 nm laser irradiation at 1.1 W/cm^2^ for 5 min. The temperature at the tumor tissue targeted by CLB-ZW800 1 h post injection rapidly increased up to ~54 °C during the 5 min of laser irradiation, while the tumor temperatures injected with PBS alone or with ZW800-1 displayed small changes (37.7 and 42.4 °C) under the same condition (Figure 6a). This demonstrates that the increased temperature at the tumor site was generated by the photothermal conversion effect of CLB-ZW800. The CLB-ZW800-treated tumors showed a phototherapeutic temperature close to 50 °C at 2 min post irradiation, then the tumor temperature increased up to ~54 °C for the next 3 min of laser irradiation (Figure 6b). Since the increased tumor temperature produced by CLB-ZW800 is sufficient to induce the apoptosis of cancer cells, CLB-ZW800 can be successfully utilized in both tumor-targeted imaging and effective photothermal cancer therapy.

### 2.5. In Vivo Photothermal Therapeutic Efficacy

**Figure 7:**
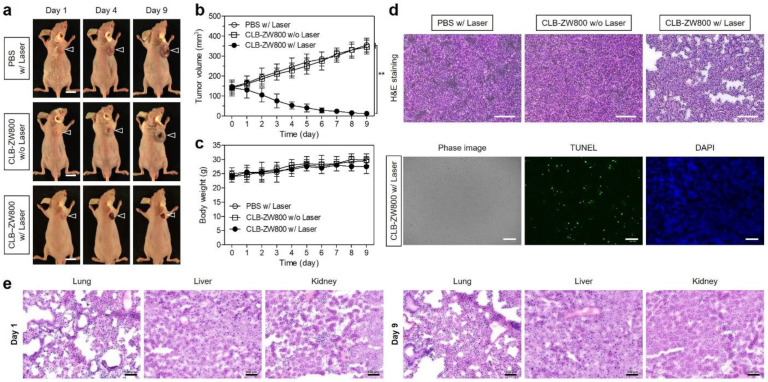
In vivo NIR phototherapeutic efficacy. (**a**) Representative photos of tumor size changes in HT-29 tumor-bearing mice for 9 days after different treatments. The laser groups were treated at 1 h post injection with PBS or CLB-ZW800, followed by 808 nm laser irradiation (1.1 W/cm^2^) for 5 min. The tumor sites are indicated by arrowheads. Scale bars = 1 cm. (**b**) Tumor growth rates and (**c**) body weights of each treatment group were monitored for 9 days. Data are expressed as mean ± S.D. (*n* = 3); ** *p* < 0.01. (**d**) Tumor sections stained with H&E from each group after 24 h of different treatments (top panel). TUNEL (green) and DAPI (blue) staining of the tumor section treated with CLB-ZW800 and laser irradiation (bottom panel). (**e**) H&E stained images of major organs, including lungs, liver, and kidneys, after 1 and 9 days of CLB-ZW800 treatment alone, respectively. Scale bars = 100 μm. Images are representative of N = 3 independent experiments.

To confirm the phototherapeutic effect of CLB-ZW800, the HT-29 tumor-bearing mice in each treatment group were monitored for 9 days after PTT (Figure 7a). The tumors injected with CLB-ZW800, without laser irradiation, showed similar growth rates to those of tumors treated with PBS followed by laser irradiation. This indicates that there were no therapeutic effects of CLB-ZW800 alone, owing to its short retention time in the tumor tissue, or laser irradiation alone without the PTT agent. As expected, the mice group injected with CLB-ZW800 followed by laser irradiation exhibited an obvious decrease in tumor volume within 5 days, and finally, only the black scars remained (Figure 7b). This suggests that CLB-ZW800, armed with laser irradiation, can effectively suppress tumor growth without recurrence or treatment-induced toxicity. Moreover, no significant change in body weight was confirmed in the mice group treated with CLB-ZW800 and laser irradiation during the course of the treatment, indicating high biosafety of the PTT system and good biocompatibility of CLB-ZW800 (Figure 7c). Finally, the tumors were collected from each group at 24 h after different treatments and examined using H&E staining for histological analysis. Clear evidence of the morphological patterns of cell damage, including cell shrinkage, necrotic cell death, and reduced cell area, were observed in tumors treated with CLB-ZW800 and laser irradiation, whereas the tumors in the other treatment groups showed normal patterns of cell proliferation, without any therapeutic effects. More evidently, the apoptotic cells in the tumors treated with CLB-ZW800 and laser irradiation were reconfirmed by the terminal deoxynucleotide transferase-mediated dUTP nick end labeling (TUNEL) assay, denoted by green fluorescence signals (Figure 7d). Additionally, histological assessment of the major organs, including lungs, liver, and kidneys resected from the CLB-ZW800 alone treatment group, revealed no pathological changes or lesions (Figure 7e). Therefore, this result demonstrates that the CLB-ZW800 conjugate could be used for the effective in vivo PTT application, with high phototherapeutic efficacy.

## 3. Discussion

In the present study, a highly water-soluble zwitterionic NIR fluorophore was synthesized to conjugate with a hydrophobic and cytotoxic anticancer drug CLB to improve tumor targeting, in vivo biodistribution, and PTT performance. In vivo results of the CLB-ZW800 conjugate revealed high tumor uptake and rapid body clearance within 4 h after injection. Because of the high cytotoxicity of CLB, it is important that the CLB-ZW800 conjugate could be rapidly eliminated from the body to prevent adverse side effects induced by nonspecific tissue/organ uptake. Thus, this study confirmed that the CLB-ZW800 conjugate preserved the unique in vivo performance of the zwitterionic NIR fluorophore ZW800 and could provide clues for utilizing various kinds of hydrophobic and cytotoxic drugs. Furthermore, the tumor tissue targeted by the CLB-ZW800 conjugate was treated with a 808 nm NIR laser for noninvasive photothermal ablation therapy because of the excellent photothermal conversion property of the ZW800 NIR fluorophore.

Although the CLB-ZW800 conjugate shows the possibility for future as a tumor-targeting phototherapeutic agent in the field of hydrophobic anticancer drug delivery and phototherapy, several problems still remain for the creation of the anticancer effect of the CLB-ZW800 conjugate without the additional photothermal treatment in tumor-bearing mice. Indeed, there is no choice but to use the PTT system in this study to produce the significant anticancer activity of CLB because of the short retention time of CLB-ZW800 in the tumor tissue. In this regard, macromolecular drug carriers, such as organic or inorganic nanoparticles consisting of various polymers, proteins, and metals, may be employed for the prolonged retention time of CLB-ZW800 in the tumor tissue to maximize the anticancer effect of CLB. Alternatively, the conjugation strategy of CLB-ZW800 may be reconsidered to allow the CLB moiety to remain in the tumor microenvironment through the reactive oxygen species (ROS)-triggered activation process using a cleavable disulfide linker. Nevertheless, the ROS-responsive prodrug system may not be suitable for the PTT applications due to the separation of the conjugated NIR fluorophore.

To highlight the advantages of CLB-ZW800, in this study, the poor tumor-selective CLB and non-tumor-targetable zwitterionic fluorophore could create a significant synergistic effect to act as a tumor-targeting agent, as well as an effective PTT agent after conjugation. This study is the first report to develop the tumor-targeted small-molecule PTT agent by using the highly hydrophobic anticancer drug CLB and the highly water-soluble zwitterionic NIR fluorophore. Considering ongoing issues of macromolecular drug carriers, including complicated synthetic processes and unsolved biological safety, this simple and effective method could provide a clue for designing the target-specific imaging agents for the use of hydrophobic small molecule drugs.

## 4. Materials and Methods

### 4.1. Conjugation of Chlorambucil to the Zwitterionic NIR Fluorophore (CLB-ZW800)

All reagents and solvents were purchased from Sigma-Aldrich (St. Louis, MO, USA) and were used without further purification. The ZW800-Cl heptamethine cyanine fluorophore was prepared as described previously [11]. Before introducing a tyramine linkage on the meso-chlorine atom of ZW800-Cl, tert-butyloxycarbonyl (Boc)-protected tyramine was prepared by adding triethylamine (0.23 g, 2.28 mmol) and Boc anhydride (0.5 g, 2.29 mmol) into the tyramine solution (0.21 g, 1.53 mmol) in dimethylformamide (DMF; 5 mL). The reaction mixture was stirred at ambient temperature for 2 h. To the above solution, under nitrogen atmosphere, sodium hydride (0.04 g, 1.6 mmol) was added, and the mixture was stirred at ambient temperature for 1 h. Subsequently, ZW800-Cl (0.1 g, 0.12 mmol) was added to the above solution, and the mixture was stirred at ambient temperature for 17 h. For the Boc deprotection, a solution of trifluoroacetic acid (TFA) and water (5 mL, 50/50 *v*/*v*%) was mixed with the above solution and stirred at ambient temperature for additional 2 h. The crude mixture was crystallized with ethyl acetate, collected, and dried under vacuum. The crude product was separated using a preparative high-performance liquid chromatography (HPLC) system (Waters, Milford, MA, USA) to obtain the amine-functionalized zwitterionic NIR fluorophore (ZW800-AM). Finally, chlorambucil (6 mg, 0.02 mmol) was conjugated to ZW800-AM (10 mg, 0.01 mmol) in the presence of 4-(4,6-dimethoxy-1,3,5-triazin-2-yl)-4-methylmorpholinium chloride (DMT-MM; 5 mg, 0.02 mmol) in dimethyl sulfoxide (DMSO; 5 mL) at ambient temperature for 12 h. The crude product was repeatedly separated using a preparative HPLC system (Waters). The molecular weights of the purified ZW800-AM and CLB-ZW800 were confirmed by the Dionex UltiMateTM 3000 mass spectrometry system (Thermo Scientific, Waltham, MA, USA).

### 4.2. Optical and Physicochemical Property Analyses

Optical properties of CLB-ZW800 were measured in phosphate-buffered saline (PBS, pH 7.4). The absorption spectrum of CLB-ZW800 was measured using a fiber optic UV-Vis-NIR (200–1025 nm) spectrometer (Ocean Optics, Dunedin, FL, USA). The molar extinction coefficient (*ε*) was calculated using the Beer–Lambert equation. The fluorescence emission spectrum of CLB-ZW800 was recorded using a SPARK^®^ 10M microplate reader (Tecan, Männedorf, Switzerland) at an excitation wavelength of 720 nm and emission wavelengths ranging from 770 to 900 nm. In silico predictions of the distribution coefficient (log*D* at pH 7.4) and topological polar surface area (TPSA) were performed using Marvin and JChem calculator plugins (ChemAxon, Budapest, Hungary).

### 4.3. In Vitro Cell Binding and NIR Fluorescence Microscopy

The human colorectal adenocarcinoma cell line HT-29 was obtained from the American Type Culture Collection (ATCC, Manassas, VA, USA). Cancer cells were maintained in Roswell Park Memorial Institute (RPMI) 1640 medium (Gibco BRL, Paisley, UK) supplemented with a 10% fetal bovine serum (FBS, Gibco BRL) and an antibiotic-antimycotic solution (Welgene, Daegu, Republic of Korea) in a humidified 5% CO_2_ atmosphere at 37 °C. When the cells reached a confluence of approximately 50%, they were rinsed twice with PBS and the CLB-ZW800 was added to each well at various concentrations in the range of 2–20 μM. The HT-29 cells were incubated for 24 h at 37 °C and then gently washed with PBS. NIR fluorescence imaging was performed using a Nikon Eclipse Ti-U inverted microscope system (Nikon, Seoul, Republic of Korea). Image acquisition and analysis were performed using the NIS-Elements Basic Research software (Nikon).

### 4.4. In Vitro Cytotoxicity Assay

Cell toxicity and proliferation were evaluated using a 3-(4,5-dimethylthiazol-2-yl)-2,5-diphenyltetrazolium bromide (MTT, Sigma-Aldrich) assay. The HT-29 cells were seeded onto 96-well plates (1 × 10^4^ cells per well). To evaluate the cytotoxicity, depending on the CLB-ZW800 concentration, the cancer cells were treated with the CLB-ZW800 conjugate (2, 5, 10, 20, and 50 μM) for 1 h and cultured for 24 h after treatment. At each time point, the incubation cell medium was replaced with 100 μL of fresh medium, and 10 μL of the MTT solution was directly added to each 100 μL well. Subsequently, the plates were then incubated for 4 h at 37 °C in a humidified 5% CO_2_ incubator. Finally, the plates were placed in a microplate reader (SPARK^®^ 10M, Tecan) to measure the absorption intensity at 570 nm. Cell viability was calculated using the following formula: cell viability (%) = (*A*_sample_ − *A*_blank_)/(*A*_control_ − *A*_blank_) × 100, where *A* is the average absorbance.

### 4.5. HT-29 Xenograft Mouse Model

Animal studies were performed in accordance with the guidelines approved by the Chonnam National University Animal Research Committee (CNU IACUC-H-2020-19). Adult (6-week-old, ≈25 g) male athymic nude mice (N = 3 independent experiments) were purchased from OrientBio (Gwangju, Republic of Korea). HT-29 cancer cells were cultured and suspended in 100 μL of PBS before being subcutaneously inoculated in the right flank of each mouse (1 × 10^6^ cells per mouse). When tumor sizes reached about 1 cm in diameter, CLB-ZW800 was administered intravenously. Animals were euthanized for in vivo NIR fluorescence imaging within a designated period of time.

### 4.6. In Vivo Biodistribution and Tumor Imaging

In vivo NIR fluorescence imaging was performed using a FOBI imaging system (NeoScience, Suwon, Republic of Korea). The mice were sacrificed 4 h after injection, and their main organs (heart, lungs, liver, pancreas, spleen, kidneys, duodenum, and intestine) were harvested and imaged to confirm the time-dependent biodistribution of CLB-ZW800. The fluorescence intensities of the tumors and excised organs were analyzed using ImageJ software (National Institutes of Health, Bethesda, MD, USA).

### 4.7. In Vivo Photothermal Therapeutic Efficacy

CLB-ZW800 or PBS were intravenously injected into the HT-29 tumor-bearing mice, and the mice were anaesthetized after 1 h. The tumors were treated with a laser (1.1 W/cm^2^, *λ* = 808 nm) for 5 min. Temperature changes at the tumor sites were monitored using a thermal imager (FLIR Systems, Wilsonville, OR, USA). Tumors were excised from the treated mice 24 h after irradiation for subsequent analysis of histological samples stained with hematoxylin and eosin (H&E). To assess the in vivo antitumor effect, the macroscopic tumor growth of each group was observed for 9 days. The tumor volume (V) was measured by the following formula: V = 0.5 × longest diameter × (shortest diameter)^2^.

### 4.8. Statistical Analysis

Statistical analysis was performed by one-way analysis of variance for a multiple comparison test. The results were represented as mean ± standard deviation (S.D.). A value of *p* < 0.05 was considered statistically significant. Curve fitting was performed using Prism software (GraphPad, San Diego, CA, USA).

### 4.9. Histological Analysis

Resected tumors were preserved for H&E staining and microscopic observation. The tumors were fixed in 4% paraformaldehyde and flash-frozen in an optimal cutting temperature (OCT) compound using liquid nitrogen. Frozen samples were cryosectioned (10 µm thick), stained with H&E, and observed using a microscope. Histological analysis was performed on a Nikon Eclipse Ti-U inverted microscope system (Nikon).

### 4.10. TUNEL Assay

Tumor tissues were collected from the CLB-ZW800 and laser-treated group to confirm an observation of apoptotic cell death. They were fixed in 4% paraformaldehyde at −20 °C for 30 min and cryosectioned (10 µm in thickness per slide). Then, the samples were washed with PBS and incubated with a terminal deoxynucleotide transferase-mediated dUTP nick end labeling (TUNEL) reagent containing terminal deoxynucleotidyl transferase and fluorescent isothiocyanate dUTP using a DeadEnd^TM^ Fluorometric TUNEL System (Promega, Madison, WI, USA). After incubation, they were stained with 4′,6-diamidino-2-phenylindole (DAPI; 1 μg/mL) for 30 min to investigate the cell nucleus by UV light microscopic observations (blue). Fluorescence imaging was performed on a Nikon Eclipse Ti-U inverted microscope system. Image acquisition and analysis were performed using NIS-Elements Basic Research software (Nikon).

## Data Availability

Not applicable.
